# Risk factors associated with cognitive frailty among community-dwelling older adults: A scoping review

**DOI:** 10.1093/geroni/igab046.3007

**Published:** 2021-12-17

**Authors:** Sumi Lee, JuHee Lee

**Affiliations:** Yonsei University, Seoul, Seoul-t'ukpyolsi, Republic of Korea

## Abstract

Objectives: This study aimed to explore the risk factors associated with cognitive frailty(CF) among community-dwelling older adults, and to provide the impact of CF on health-related outcomes. Methods: PubMed, EMBASE, Cochrane, PsycINFO, CINAHL, RISS, DBpia, NDSL, and KoreaMed databases were searched to retrieve studies. Two reviewers independently screened titles, abstracts and articles. The inclusion criteria are peer-reviewed articles written in English or Korean for community-dwelling older adults with both physical frailty and cognitive impairment present at the same time. Results: A total of 3,513 were searched, and the final 33 were extracted according to the inclusion criteria. Physical factors affecting CF were the number of chronic disease, cardiovascular disease, activity of daily living(ADL), making telephone calls and shopping during instrumental ADL, and a Mini Nutrition Assessment–Short Form score. Psychological factor was depressive symptoms. Significant behavioral factors included self-reported physical activity, low vitamin D, smoking, frequent insomnia, and sedentary lifestyle. In social factors, social participation such as volunteering was identified as a protective factor. Mortality, followed by dementia was health related outcomes on CF, including ADL dependence, poor quality of life, and hospitalization. However, the CF-related fall was inconsistent. Conclusion: A wide variety of factors have been presented in studies related to CF. In order to understand CF and improve health-related outcomes, older adults in CF should be screened as high-risk group. When the risk factors and protective factors of CF managed, better health-related outcomes will lead to successful aging of community-dwelling older adults.

